# Influenza A Virus with a Human-Like N2 Gene Is Circulating in Pigs

**DOI:** 10.1128/genomeA.00712-13

**Published:** 2013-09-19

**Authors:** Solvej Østergaard Breum, Charlotte Kristiane Hjulsager, Ramona Trebbien, Lars Erik Larsen

**Affiliations:** Section of Virology, Technical University of Denmark, National Veterinary Institute, Frederiksberg C, Denmark

## Abstract

A novel reassortant influenza A virus, H1avN2hu, has been found in Danish swine. The virus contains an H1 gene similar to the hemagglutinin (HA) gene of H1N1 avian-like swine viruses and an N2 gene most closely related to the neuraminidase (NA) gene of human H3N2 viruses from the mid-1990s.

## GENOME ANNOUNCEMENT

Influenza A viruses are important human and animal pathogens with high impacts on public and animal health. These viruses contain eight separate RNA segments, and the hemagglutinin (HA) and neuraminidase (NA) segments define the subtype. In the case of a mixed infection by two viral strains within a cell, the exchange of gene segments may lead to a new reassortant virus. The influenza A viruses of the H1N1, H1N2, and H3N2 subtypes are enzootic in swine. The origin, and therefore the antigenic and genetic characteristics of these subtypes, differ between continents and geographic regions. New swine influenza virus reassortants originating from precursor avian, human, and/or swine viruses continue to emerge (1–3).

In Denmark, a passive surveillance program for influenza A virus has been performed since 2011 using samples from pigs with acute respiratory diseases submitted for diagnostic purposes. A selection of positive samples were subtyped by partial sequencing of the HA and NA genes, which revealed the presence of a new reassortant influenza virus subtype in several of the submissions. From three of these submissions, virus was isolated in cell cultures and the new subtype was confirmed by sequencing. Full-genome sequencing was performed for one virus isolate by full-length amplification of all 8 gene segments with in-house-designed primers using the SuperScript III OneStep reverse transcriptase PCR (RT-PCR) system with Platinum *Taq* High Fidelity. Purified PCR products were pooled in equimolar quantities and used for next-generation sequencing on the Ion Torrent PGM sequencer.

Phylogenetic analyses of the obtained genome sequence showed that all genes, except for the N2 gene, were highly similar to H1N1 avian-like viruses, which have been circulating in the Danish pig population for at least three decades. Interestingly, the N2 gene was approximately 94% identical at both the nucleotide and amino acid levels to N2 of the seasonally human H3N2 viruses from the mid-1990s. Virus carrying this new subtype, H1avN2hu, was found in two or three submissions from different herds in each of the years 2011 to 2013, indicating that it may already be established in the Danish pig population. Furthermore, influenza A viruses carrying a similar human-like N2 gene but in a pandemic H1N1pdm09 backbone were detected in swine in Germany in 2011 (4); these viruses have also been detected in a few submissions since 2011 in Denmark (S. Ø. Breum, C. K. Hjulsager, R. Trebbien, and L. E. Larsen, unpublished data). The results confirm that continued surveillance of influenza A viruses in animals and humans is essential since the virus can adapt or swap genes within a number of species, including pigs, potentially resulting in mammalian-adapted influenza A viruses with zoonotic potential.

### Nucleotide sequence accession numbers.

The complete genome sequence of strain A/swine/Denmark/10302-2/2012(H1N2) has been deposited in GenBank under the accession no. KF514376 to KF514383 for segments 1 to 8.

## References

[1] Howard WA Essen SC Strugnell BW Russell C Barass L Reid SM Brown IH 2011 Reassortant pandemic (H1N1) 2009 virus in pigs, United Kingdom. Emerg. Infect. Dis. 17:1049–1052 21749767 10.3201/eid1706.101886 PMC3358214

[2] Kitikoon P Nelson MI Killian ML Anderson TK Koster L Culhane MR Vincent AL 2013 Genotype patterns of contemporary reassorted H3N2 virus in U.S. swine. J. Gen. Virol. 94:1236–1241 23695819 10.1099/vir.0.51839-0

[3] Moreno A Di Trani L Faccini S Vaccari G Nigrelli D Boniotti MB Falcone E Boni A Chiapponi C Sozzi E Cordioli P 2011 Novel H1N2 swine influenza reassortant strain in pigs derived from the pandemic H1N1/2009 virus. Vet. Microbiol. 149:472–477 21208754 10.1016/j.vetmic.2010.12.011

[4] Starick E Lange E Grund C Grosse Beilage E Döhring S Maas A Noé T Beer M Harder TC 2012 Reassortants of pandemic influenza A virus H1N1/2009 and endemic porcine HxN2 viruses emerge in swine populations in Germany. J. Gen. Virol. 93:1658–1663 22622326 10.1099/vir.0.042648-0

